# Objectives, Outcomes, Facilitators, and Barriers of Telemedicine Systems for Patients with Alzheimer’s Disease and their Caregivers and Care Providers: A Systematic Review

**DOI:** 10.34172/aim.2022.90

**Published:** 2022-08-01

**Authors:** Parasto Amiri, Zahra Niazkhani, Habibollah Pirnejad, Mahdie ShojaeiBaghini, Kambiz Bahaadinbeigy

**Affiliations:** ^1^Student Research Committee, Kerman University of Medical Sciences, Kerman, Iran; ^2^Nephrology and Kidney Transplant Research Center, Clinical Research Institute, Urmia University of Medical Sciences, Urmia, Iran; ^3^Patient Safety Research Center, Clinical Research Institute, Urmia University of Medical Sciences, Urmia, Iran; ^4^Erasmus School of Health Policy and Management, Erasmus University Rotterdam, Rotterdam, The Netherlands; ^5^Medical Informatics Research Center, Institute for Futures Studies in Health, Kerman University of Medical Sciences, Kerman, Iran

**Keywords:** Alzheimer’s disease, Barriers, Caregivers, Drivers, Telemedicine, Systematic review

## Abstract

**Background:**

Alzheimer’s disease is an extremely expensive chronic disease, which is rapidly becoming a major cause of mortality in adults. For over two decades, telemedicine has been used to assist patients and their caregivers to manage this disease. The present study aimed to evaluate the objectives, outcomes, facilitators, and barriers influencing the use of telemedicine systems for patients with Alzheimer’s disease and their caregivers and care providers.

**Methods:**

In this systematic review, we searched for the original articles published in databases such as PubMed, Web of Science, and Scopus until November 2021 using relevant keywords. A qualitative content analysis was performed the based on the theory of planned behavior and the health belief model using the ATLAS.ti software.

**Results:**

In total, 1191 articles were identified, and 60 articles were included in this study. While having different objectives, most of the studies compared telemedicine systems to in-person visits (21.43%) and assessed the feasibility of the implemented method (16.07%). The overall outcomes of telemedicine in the articles were classified as cost-effectiveness (e.g., reduced commute, fuel, and time to access care), clinical outcomes (e.g., lower anxiety, stress, and depression), and patient, caregiver, and healthcare provider satisfaction. In total, 19 facilitators and 12 barriers influencing the use of telemedicine for patients with Alzheimer’s disease and their caregivers were identified.

**Conclusion:**

According to the results, telemedicine systems could be implemented for various reasons. Developing a clear framework of the drivers and barriers before the implementation of these systems could improve decision-making prior to the design and implementation of telemedicine systems.

## Introduction

 In the 21^st^ century, aging is associated with multiple age-specific diseases, such as dementia.^1^ Studies show that about 11% of the individuals aged more than 65 years are diagnosed with dementia throughout the world, and more than one-third of the people aged more than 85 years tend to experience this condition.^2^ In a report by the World Health Organization (WHO) in 2008, dementia was recognized as a priority condition in the WHO mental health plan.^3^ Based on the prediction of the WHO in 2015, the number of the patients with dementia will reach 74 million in 2030.^1^

 Alzheimer’s disease is the most common cause of dementia,^4^ and the WHO has predicted that Alzheimer’s disease will become the fourth cause of mortality in the world by 2050.^5^ With disease progression, patients with Alzheimer’s disease become unable to take care of their daily affairs alone.^6^ Consequently, they require a greater level of care at the advanced stages of the disease, which is time-consuming and costly.^7^

 According to statistics, the costs imposed by Alzheimer’s disease would increase throughout Europe from $177.2 billion to over $250 billion during 2008–2030.^7,8^ Today, more than 16 million family members provide an estimated 18.6 billion hours of care to patients with Alzheimer’s disease.^9^ In addition to the high costs and waste of time for care of patients with Alzheimer’s disease, physical and psychological problems have been detected in their caregivers, which are also important health concerns. Therefore, the caregivers of patients with Alzheimer’s disease require constant support to prevent the increased prevalence of depression, anxiety, and occupational burnouts from the moment of diagnosis until the patients’ death.^6,10^

 Given the need for the continuous care of patients with Alzheimer’s disease by the caregivers, barriers such as the unavailability of specialists and medical facilities in remote areas, and the time and costs of transporting the patients to the nearest health center/hospital, supporting these caregivers in-person and by conventional methods is extremely complicated and even impossible.

 Today, technology could be used to transfer support and care to the most remote areas. For instance, telemedicine contributes to the support of patients with Alzheimer’s disease and their caregivers. Recently, wide-ranging medical services could be provided in hospitals, outpatient wards, and physician offices through telemedicine. The WHO telemedicine policy in relation to the development of the health-for-all strategy was defined and developed in 1997.^11^ Accordingly, telemedicine is defined as the delivery of healthcare services where distance is a critical factor by all healthcare professionals using information and communication technologies for the exchange of valid information for the diagnosis, treatment, and prevention of diseases and injuries, as well as research and evaluation and for the continuous education of the healthcare providers in the interest of advancing the health of individuals and their communities.

 Recently, telemedicine has been established and considered in numerous areas in developed and developing countries in order to support patients with Alzheimer’s disease and their caregivers.^12-14^ In a research conducted in the United States (2010), telemedicine was identified as a cost-effective method for patients with Alzheimer’s disease with lower costs than in-person visits.^15^ Moreover, telemedicine has been shown to increase patient satisfaction through reducing their stress and anxiety, while also decreasing the caregivers’ workload as there would be no need for the displacement of patients for an in-person visit.^16^ Given the benefits of telemedicine and the issues associated with the care of patients with Alzheimer’s disease and their caregivers, this technology is expected to effectively help these individuals. Despite the positive impacts of telemedicine on patients, some challenges have been attributed to this approach, as well.

 In a survey performed in 2012, cultural, managerial, technical, and monitoring problems were among the barriers to telemedicine implementation.^17^ In some studies, technical obstacles have been reported to significantly affect the establishment or use of telemedicine systems.^9,11,13,18-21^ Furthermore, professional, legal, and financial obstacles have been highlighted in this regard, with legal and financial barriers known to be of a managerial nature.^22^ In developed and developing countries, these barriers may prevent rapid expansion of telemedicine in healthcare environments.

 In addition to the mentioned issues, developing countries experience communication infrastructure problems (e.g., poor internet speed) in the establishment of these systems.^23^ Several studies have systematically assessed the barriers to telemedicine in some countries.^24-26^ To the best of our knowledge, some systematic studies have also focused on telemedicine for patients with Alzheimer’s disease and their caregivers, while none has assessed the drivers and barriers to these systems.^27,28^ With this background, the present study aimed to systematically review the outcomes and objectives of the studies on the use of telemedicine for patients with Alzheimer’s disease and their caregivers, and the second section of the article is dedicated to the drivers and barriers influencing the development and implementation of this method.

## Materials and Methods

 This systematic review was conducted in accordance with the guidelines of the Preferred Reporting Items for Systematic Reviews and Meta-analyses (PRISMA).^29^

###  Search Strategy 

 A literature search was performed in databases such as Scopus, PubMed, and Web of Science for the relevant articles published in English until November 2021. We used various keyword combinations in the titles and abstracts of the articles, including (Alzheimer’s disease) OR (dementia) OR (cognitive impairment)) AND (telemedicine) OR (tele-medicine) OR (telehealth) OR (Tele-health) OR (telecare) OR (telemonitoring) OR (telehomecare) OR (remote monitoring) OR (teleconsult) OR (remote consult)). Table 1 shows the complete search strategy.

**Table 1:** Search Strategies in PubMed, Scopus, and Web of Science Databases

**Database**	**Search Strategies**
PubMed	(((Alzheimer's Disease [MeSH]) OR (dementia [MeSH])) OR (cognitive impairment [Title/Abstract])) AND (((((((((((Telemedicine [MeSH]) OR (Tele-medicine [Title/Abstract])) OR (Telehealth [Title/Abstract])) OR (Tele-health [Title/Abstract])) OR (Telecare [Title/Abstract])) OR (Telemonitoring [Title/Abstract])) OR (Telehomecare [Title/Abstract])) OR (Remote monitoring [Title/Abstract])) OR (teleconsult [Title/Abstract])) OR (remote consult [Title/Abstract])))
Scopus	( ( TITLE-ABS-KEY ( "Alzheimer's Disease" ) OR TITLE-ABS-KEY ( dementia ) OR TITLE-ABS-KEY ( "cognitive impairment" ) ) ) AND ( ( TITLE-ABS-KEY ( telemedicine ) OR TITLE-ABS-KEY ( "Tele-medicine" ) OR TITLE-ABS-KEY ( telehealth ) OR TITLE-ABS-KEY ( "Tele-health" ) OR TITLE-ABS-KEY ( telecare ) OR TITLE-ABS-KEY ( telemonitoring ) OR TITLE-ABS-KEY ( telehomecare ) OR TITLE-ABS-KEY ( "Remote monitoring" ) OR TITLE-ABS-KEY ( teleconsult ) OR TITLE-ABS-KEY ( "remote consult" ) ) ) )
Web of Science	1: TS = ((Alzheimer's Disease) OR dementia OR (cognitive impairment)) 2: TS = (Telemedicine OR (Tele-medicine) OR Telehealth OR (Tele-health) OR Telecare OR Telemonitoring OR Telehomecare OR (Remote monitoring) OR teleconsult OR (remote consult)) #1 AND #2

###  Inclusion and Exclusion Criteria

 Interventional and observational studies regarding the use of telemedicine services for patients with Alzheimer’s disease were included in the study. Studies were excluded if they were not focused on Alzheimer’s disease, along with review articles, opinion papers, editorials, letters to the editor, and the abstracts presented in a conference.

###  Review Process 

Figure 1 shows the flow diagram of the review process. Three authors independently screened the titles and abstracts of the articles to find other relevant studies based on the inclusion and exclusion criteria. Relevant articles were selected for a full-text review, and disagreements were resolved by consensus. A data list of the selected full-text articles was generated in the Excel Spreadsheet software version 2018.

**Figure 1:**
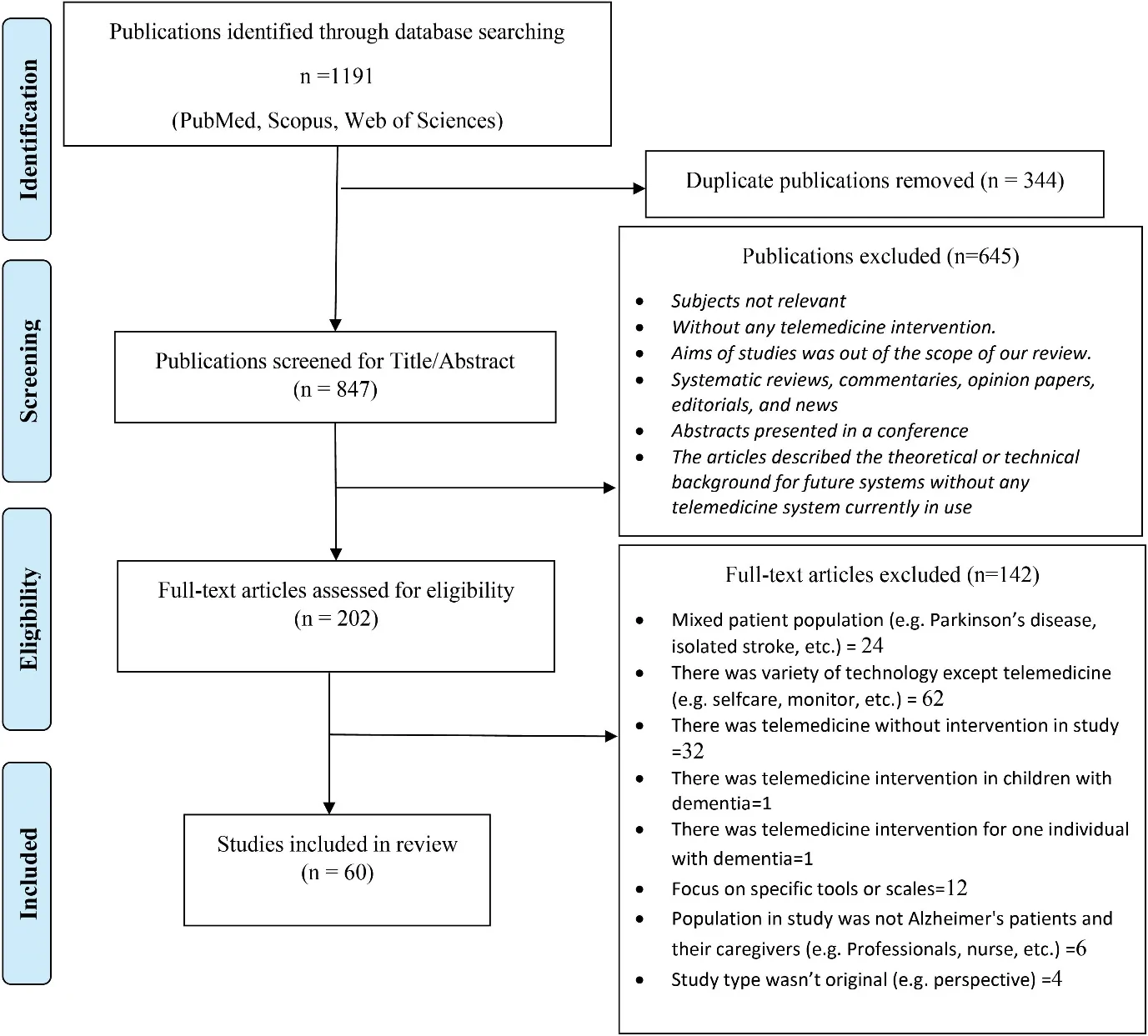
Flow Diagram of Article Selection

###  Data Extraction and Analysis 

 In the present study, we identified the drivers and barriers influencing the application of telemedicine systems using qualitative content analysis in the ATLAS.ti software version 8.4.24.^30^ The researchers defined the drivers and barriers to telemedicine use based on the theory of planned behavior and the health belief model. The theory of planned behavior presents a model on human behavior guidance, showing the individual’s intention to perform a specific behavior under the influence of internal constraints (character, knowledge, skills and abilities, feelings) and external constraints (time, position, behavior of others) on showing a specific behavior.^31^ Based on the health belief model, individuals often respond well when their health is endangered (perceived susceptibility) or when a situation involves a high risk (perceived severity), and behavior change works to their benefit (perceived benefits). In addition, they attempt to eliminate issues such as high costs, waste of time, and discomfort, which may prevent healthy activities (perceived barriers), so that the individual could successfully take a health-related measure (self-efficacy).^32,33^ Based on this definition, drivers and barriers are divided into two internal and external sets; internal drivers refer to the positive feedback, comments, factors, and indicators that affect the behavior and motivation of the individual while using the system. External drivers include the positive feedback, opinions, factors, and indicators regarding the surrounding environment of applying the system and the system itself. On the other hand, internal barriers may refer to the negative feedback, opinions, factors, and indicators that affect the behavior and motivation of the individual while using the system, and external barriers could be considered the negative feedback, opinions, factors, and indicators regarding the surrounding environment of applying the system and the system itself.

 After extracting the drivers and barriers influencing telemedicine from the full-text of the articles, several group sessions were held to discuss the discovered data and themes and reach a consensus. Moreover, the authors extracted the basic features of each study, summarized the research objectives, and described the telemedicine systems used in each study. The effectiveness of the telemedicine systems used in the studies was summarized, as well.

## Results

 In total, 1191 articles were retrieved, and the number was reduced to 847 articles after eliminating duplicate publications, titles, and abstracts. Finally, the full texts of 202 out of 847 articles were reviewed. Table S1 shows the data of 60 articles (Supplementary file 1).

###  Data of the Selected Studies 

 Table S1 shows the retrieved articles and their year of publication (1998–2021). Accordingly, most of the articles (n = 10; 16.7%) were conducted in 2018 and in the United States. One study was published in the form of a joint article between the United States and the United Kingdom,^34^ and four other papers (6.7%) were jointly performed by several countries.^35-38^ Among these, two cases were not included due to the multiplicity of the countries.^35,36^ Notably, only five articles (8.3%) were published in Asian countries (China, Taiwan, and South Korea).^14,39-42^

 In the present study, 50, seven, and three of 60 articles were quantitative, qualitative, and mixed, respectively (Table S1). Moreover, 29 of the quantitative articles (48.3%) were randomized clinical trials. Most of the studies (n = 47; 78.3%) were performed in affiliation with an academic center. In 34 studies (56.7%), the patients or their caregivers received medical services at home. In 26 studies (43.3%), health services were provided to the patients and their caregivers at equipped hospital rooms, rural clinics, outpatient clinics, universities, and healthcare centers.

###  Participants

 In the reviewed studies, the participants included patients with Alzheimer’s disease (60%), patients with cognitive disorders (13.3%), their caregivers (50%), and healthcare providers (5%). One of the studies recorded the number of phone calls rather than the number of participants.^43^ According to our findings, the target population of 24 studies (40%) was patients with Alzheimer’s disease and healthcare providers, which mainly focused on the support for caregivers. Moreover, 12 studies were similar in terms of the patients, their caregivers, and healthcare providers, and their objective was to assess the support for both groups (patients with Alzheimer’s disease and their caregivers).

###  Duration of the Studies

 The duration of the reviewed studies differed in terms of weeks, months, and years. The shortest period was two weeks,^44,45^ and the longest period was 10–20 years.^46^ Notably, the duration of the study was not mentioned in five articles.^12,37,47-49^

###  Type of Caregivers 

 Two types of caregivers were mentioned in the reviewed studies (family caregiver and health staff care provider). In 60 studies, spouses (30%) and adults (19.6%) constituted the majority of the caregivers. Two studies reported the caregivers to be the family members of the patients without specifically mentioning their kinship.^38,50^ Assuming spouses and adult children as family members, about 60% of the caregivers of the patients with Alzheimer’s disease are their family members. Approximately 9% of caregivers were health staff care provider.

###  Intervention Technologies 

 Various technologies were applied as an intervention in the reviewed studies. More than half of the studies (n = 38; 63.3%) used a video conference technology, in which videos and images are exchanged to provide healthcare services. On the other hand, telephone-based technology was used in 13 studies (21.6%) to provide and receive healthcare services. Other technologies (such as portal, website, sensor etc.) were used in 9 studies to provide and receive healthcare services. Most of the technologies applied in the articles required an internet connection for communication (76.8%), with the exception of telephone-based interventions.

###  Objectives of the Studies

 In total, 60 studies had mentioned the objectives, and three had different objectives.^42,51,52^ Ten studies assessed the feasibility of telemedicine implementation for the support of patients with Alzheimer’s disease and their caregivers. In total, 12 articles compared the effectiveness of these systems with in-person visits. In addition, most of the studies (n = 14; 23.3%) used telemedicine as a complementary technique to improve the status of patients and their caregivers. Three articles assessed and validated scales, such as the mini-mental state examination and Alzheimer’s disease assessment scale cognitive subscale, in telemedicine systems.^53-55^ The other studies had other objectives, such as design, implementation, follow-up, and evaluation.

###  Outcomes

 The outcomes of the reviewed studies were divided into three categories of cost-effectiveness, patient satisfaction, and clinical outcomes. In total, 20 out of 60 articles (33.3%) addressed the cost-effectiveness of telemedicine compared to in-person visits. According to the obtained results, the cost-effectiveness of telemedicine was due to decreased commute, fuel consumption, and time to access to care. In 18 studies (32.14%), the users of telemedicine were extremely satisfied with the system. Seven studies (12.5%) also reported improved satisfaction of patients and their caregivers, and 11 studies (19.6%) showed improved satisfaction in the patients and healthcare providers after using telemedicine services (32.14%). In fact, satisfaction with telemedicine was higher in patients, their caregivers, and healthcare providers compared to traditional visits. Eight articles (14.2%) reported increased quality of life by using telemedicine. In addition, these systems were able to partially reduce general anxiety, stress, and depression, as well as the anxiety caused by the loss/injury of the patients in their caregivers through meeting some of the basic needs of the patients and their caregivers (21.42%).

###  Drivers 

 The improvement of clinical outcomes, cost-effectiveness, and patient satisfaction were among the main drivers of telemedicine use. Other drivers were also extracted from the evaluated studies. In total, 45 articles evaluated drivers (Figure 2), the most common of which were easier access to healthcare services in remote areas through telemedicine (n = 13; 23.21%), decreased commute for in-person visits/saving time (n = 12; 21.42%), and reduced burden of the disease on the caregivers, such as bathing, clothing, and taking the patient to visits (n = 10; 17.8%).

**Figure 2:**
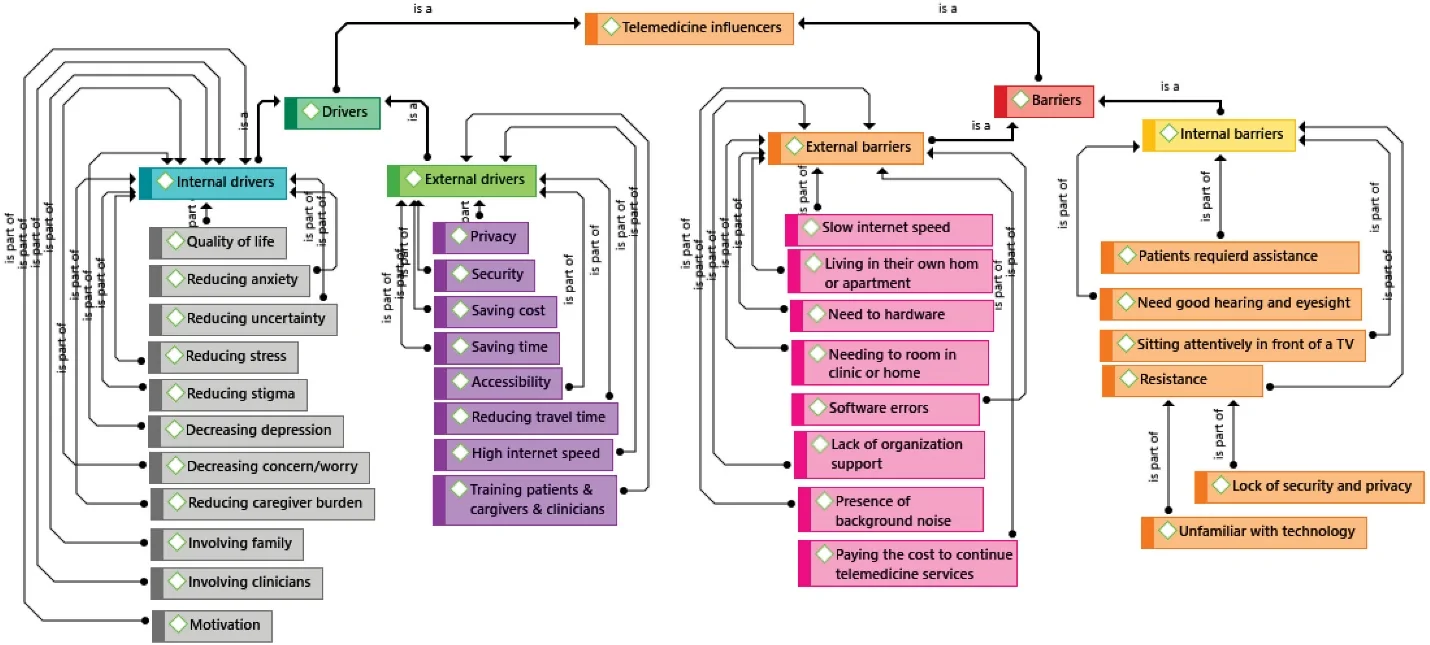
Drivers and Barriers Affecting Telemedicine Systems for Patients with Alzheimer’s Disease and their Caregivers

###  Barriers 

 In 49 studies, various barriers to the use of telemedicine systems were reported (Figure 2). Some of these factors were the need to make at least one in-person visit despite the use of telemedicine services (n = 22; 39.2%), the patient’s need for at least one companion to better interact with healthcare providers during visits (n = 9; 16%), the need for high-speed internet (n = 20; 35.7%), the need for hardware (n = 21; 37.5%), and concerns about information security and privacy (n = 11, n = 8; 19.6% and 14.3%, respectively). In these studies, the Health Insurance Portability Accountability Act was used to protect the privacy and increase the security of the patients and their caregivers.

 In addition to hardware barriers, which were frequently mentioned in the reviewed articles, software barriers were also reported in some studies. Software errors included problems related to sound echo or the short delay between image display/audio playback,^56^ noise,^47,57^ and technical issues.^43,58^ According to the results of seven studies, one of the important barriers to the successful construction of telemedicine systems was the lack of a suitable place to provide services, such as a private room in a hospital/clinic with the necessary equipment. The assessment of the articles indicated that the drivers and barriers influencing the use of telemedicine were classified as internal and external factors (Figure 2).

## Discussion

 In the present study, 60 studies were identified regarding the use of telemedicine designed for patients with Alzheimer’s disease and their caregivers. We mainly attempted to express the objectives, outcomes, drivers, and barriers affecting the design and implementation of telemedicine systems for further investigations. According to our data, a few systematic reviews focused on telemedicine systems for patients with Alzheimer’s disease and their caregivers, while no study assessed the drivers and barriers affecting the use of these systems.^27,28,59^ Notably, the systematic reviews in this regard have yielded different results. For instance, Costanzo et al only focused on the diagnostic and international outcomes of telemedicine systems in patients with Alzheimer’s disease and mild cognitive disorders up to 2018.^27^ According to the results of the mentioned study, telemedicine use had benefits such as remote diagnosis, facilitation of early diagnosis, decreased burden on the caregivers, and overcoming issues such as traveling long distances. Therefore, frequent use of these systems by patients could enhance their quality of life. Costanzo et al performed another study in 2014 to evaluate the impact of telemedicine use on improvement of the quality of life in patients with Alzheimer’s disease and their caregivers.^28^ The researcher selected articles only from the PubMed database up to 2012, and the results were indicative of the improved quality of life for the patients and their caregivers with increased use of telemedicine. Furthermore, telemedicine was reported to contribute to early diagnosis in remote areas.

 In the reviewed studies, telemedicine systems were divided into two categories of home systems (simultaneous) and clinical systems (asynchronous). Home telemedicine systems encompassed online counseling, video counseling,^60^ and telehome monitoring.^61^ These systems have been reported to provide faster access to health services compared to the clinical systems. As a result, the patients and caregivers experience less stress and anxiety about the consequences of the disease and medications since they have continuous access to health services. In addition, these patients and their caregivers have been shown to have a simpler life by using telehome monitoring, while the establishment of these systems requires a high-speed internet connection.

 The telemedicine systems give caregivers and healthcare providers multiple possibilities to create their own resources for the patients’ empowerment.

 To date, one of the goals of technology has been to improve health care and empower and improve the conditions of various patients.^62^ A key approach to achieving these goals is to access/provide remote health services^42^ as these systems could empower individuals by increasing their participation in the treatment process.^63^ Given that patients with Alzheimer’s disease are mostly old, a systematic review has recently reported that telemedicine care is currently more accessible to the elderly.^64^ However, it is still assumed that the feasibility of this method should be further evaluated in controlled randomized clinical trials since successful results of high-quality controlled randomized trials will motivate more users.

 Another quarter of the reviewed studies were classified as feasibility studies, which also compared telemedicine systems with in-person visits (controlled randomized trials). To date, several feasibility and comparative studies have focused on various diseases, including Alzheimer’s. For instance, a review conducted in 2017 showed that high-quality comparative studies have demonstrated the effectiveness of teledermatology in these patients.^65^ A few studies have also focused on the acceptability, effectiveness, feasibility, and economic aspects of these systems. A recent systematic review evaluated the effectiveness and economic aspects of telemedicine systems in emergency sections,^66^ highlighting the need for the adequate knowledge of conducting such studies. In another review (2017), telemedicine services were reported to be cost-effective in major fields of medicine, such as cardiology.^67^

 Despite the high costs of telemedicine facilities due to the need for an internet connection or purchasing video/audio communication tools and/or sensors for disease monitoring, the application of this system could reduce commuting and waiting time for visits, while increasing its own cost-effectiveness. According to the Alzheimer’s Association Report, a minimum of three in-person visits should be made for these patients per year, which may be costly for their families.^9^ In Saudi Arabia, Alaboudi et al reported the need for sustainable financial support to purchase, establish, use, and maintain the complicated infrastructures required for telemedicine.^68^ Therefore, the cost-effectiveness of this system should be further assessed in interventional studies in more details. Moreover, patient satisfaction could affect the use of these systems. In a systematic review, patient satisfaction was reported to be the most important reason for the success of telemedicine.^69^ The satisfaction of patients and their caregivers with telemedicine depends on several factors.

 In the current research, the influential factors in the increased satisfaction of patients and their caregivers with the telemedicine system included maintaining their privacy, reducing the stress caused by commute for in-person visits, reduction of the waiting time, increased access to care, visiting physicians while accompanied by family members, and decreased burden on the caregivers. Moreover, studies have reported other issues such as the reduction of readmission for surgery^70^ and contributing to the treatment plan adherence.^71^

 Another factor that could increase the motivation to use telemedicine is improved clinical outcomes. Reduced anxiety, improved quality of life, and enhanced patient care through these systems have been mentioned in several studies. Falling has long been considered a major cause of injury and increased risk of mortality in patients with Alzheimer’s disease.^72,73^ Evidence attests to the concerns regarding these patients’ fall and the possibility of injury to themselves or others^74^ since falling is also the main cause of injury and mortality in the elderly.^75,76^ According to the results of our systematic review, telemedicine systems could decrease the risk of injuries in these patients, while increasing their safety and improving their quality of life and care.

 In the current research, the drivers were classified as internal and external factors (Figure 2). Overall, we determined 11 internal drivers and eight external drivers regarding the implementation of telemedicine systems (Figure 2). Internal drivers could be beneficial to the users’ compatibility and acceptance of telemedicine systems by improving their quality of life and decreasing their stress and anxiety. On the other hand, external drivers could increase the effectiveness of the system by covering financial and security issues in the implementation and establishment of the systems. Evidently, the key role of these systems in improving the quality of life^77^ and mental health^78^ has been emphasized in several studies over decades. Evidence regarding the effectiveness of telemedicine systems in alleviating anxiety, depression, and stress is also consistent with this finding.^79,80^ Financially, the positive impact of telemedicine systems has been confirmed through saving costs and time, and the reduced number of commutes for in-person visits could also play a pivotal role in improving the quality of life of the patients. From the security perspective, health information must be protected regardless of the reason for its collection according to the HIPPA.^81^

 While the main goal of telemedicine is to reduce barriers to the use of healthcare services, our findings indicated internal and external barriers to using telemedicine systems largely influence the users. In general, we identified four main internal barriers and eight external barriers to the use of telemedicine systems (Figure 2). The most common internal barrier was the need for at least one in-person visit in the clinic/physician office, and the most frequent external barrier was the lack of access to a high-speed internet connection. Since most of the telemedicine systems used in the reviewed studies were web-based and only a few were based on phone calls, access to a high-speed internet connection had the most significant effect on the quality of non-telephone systems. In addition to the costs of purchasing proper software and hardware, the lack of a guarantee for the security and privacy of the users largely influenced the lack of acceptance of these systems.

 One of the strengths of our review study is having the maximum time interval in selecting the articles compared to similar systematic studies. In addition, a guideline or framework was presented, which contained the drivers and barriers influencing the design and implementation of telemedicine systems by researchers. One of the major limitations of the current research is the constraint in terms of selecting English articles as numerous studies in this regard have been published in non-English speaking countries, such as Korea, China, Taiwan, France, Italy, Germany, Greece, Finland, Denmark, Scotland, Rome, the Netherlands, Sweden, Iceland, and Malta. Limiting the research scope to three databases of PubMed, Scopus, and Web of Science might have led to the exclusivity of some eligible and important articles. To prevent this issue, we assessed the references of the retrieved articles, as well, in order not to eliminate relevant studies.

 According to the results, it is recommended that a clear framework of the drivers and barriers affecting the use of telemedicine systems should be used by the designers and implementers of this technique for patients with Alzheimer’s disease and their caregivers. The framework could provide the users with an overview before implementing the systems, so that better decisions could be made before attempting to design and implement these systems. For instance, lack of access to a high-speed internet connection could prevent the implementation of non-telephone telemedicine systems. Moreover, studies have mostly examined the routine of telemedicine systems to show that these systems have not replaced conventional in-person systems and have only been assistive in this regard. The comparison of telehome monitoring with clinical telemedicine systems has not been apparent either. Therefore, it is recommended that further studies evaluate and compare telehome monitoring with clinical telemedicine for patients with Alzheimer’s disease and their caregivers.

## Supplementary Materials

Supplementary file 1 contains Table S1.
