# Supplementary material for: Objectives, Outcomes, Facilitators, and Barriers of Telemedicine Systems for Patients with Alzheimer’s Disease and their Caregivers and Care Providers: A Systematic Review
Source: Arch Iran Med. 2022 Aug 1;25(8):564–73. doi: 10.34172/aim.2022.90 (PMC13661073; doi:10.34172/aim.2022.90)
Supplement: Supplementary file 1 [file aim-25-564-s001.pdf]

Arch Iran Med. August 2022;25(8):564-573
10.34172/aim.2022.90

# Objectives, Outcomes, Facilitators, and Barriers of Telemedicine Systems for Patients with Alzheimer's Disease and their Caregivers and Care Providers: A Systematic Review

**Parasto Amiri, Msc[1]; Zahra Niazkhani, MD, PhD[2]; Habibollah Pirnejad, MD, PhD[3,4]; Mahdie ShojaeiBaghini, PhD[5]; Kambiz Bahaadinbeigy, MD, PhD[5*]**

[1]Student Research Committee, Kerman University of Medical Sciences, Kerman, Iran
[2]Nephrology and Kidney Transplant Research Center, Clinical Research Institute, Urmia University of Medical Sciences, Urmia, Iran
[3]Patient Safety Research Center, Clinical Research Institute, Urmia University of Medical Sciences, Urmia, Iran
[4]Erasmus School of Health Policy and Management, Erasmus University Rotterdam, Rotterdam, The Netherlands
[5]Medical Informatics Research Center, Institute for Futures Studies in Health, Kerman University of Medical Sciences, Kerman, Iran

## Supplementary file1

**Table S1.** Characteristics of included studies in our study

| **Authors (publication year[$])** | **Country[*]** | **Type of study** | **Healthcare provider setting** | **Patient setting** | **Sample population** | **Study duration** | **Intervention participants** | **Type of caregiver(s)** | **Technology intervention** | **Objectives** | **Outcomes** | **drivers** | **Barriers** |
|---|---|---|---|---|---|---|---|---|---|---|---|---|---|
| Harvey *et al.*[1] (1998) | UK, Scotland, Ireland | Quantitative/ Cohort | Academic | Home | PwD and AD <65 and Cg (N=241) | Two years | P-P & Cg-P | Family members, general practitioner, and hospital doctor | Telephone-based | Evaluating telecare services | Cost effectiveness | Involving clinicians | N/M |
| Lee *et al.*[2] (2000) | South Korea | Quantitative/ RCT | Academic | Clinic | PwD and AD >65, Cg and Care personnel (N=140) | Two years | P-P & Cg-P | N/M | VC / VTM / CVT / VTC | Acceptability & Reliability | Cost effectiveness & Patient/provider satisfaction & Improving clinical outcomes & Improving behavioral symptoms patients | Reducing travel and saving time & Involving family & Training patients for self-administration & facilitating and supporting local services for the patient | Patients prefer face-to-face & Need internet & Software errors & Communication equipment errors & Expressing tense and frightened in the beginning |
| Mahoney *et al.*[3] (2003) | UK | Quantitative/ RCT | Academic | Home | Cg>21 (N=100, control group=51, intervention group=49) | 6, 12 and 18 months | Cg-P | Sibling, Child, Spouses | Telephone-based | Ancillary tools to empower PwD or Cg | Reducing caregivers disruptive behaviors | Reducing caregiver burden | Privacy |
| Shores *et al.*[4] (2004) | USA | Quantitative/ Cohort | Academic | Home | PwD and AD>=60 Mean age=78.7 (N=85, n=18) | Five years | P-P | N/M | VC / VTM / CVT / VTC | Diagnosis | Patient/provider satisfaction & Estimating prevalence and type of dementia | Providing services in under-served rural areas& Reducing travel and saving time & Increasing motivation | Patients prefer face-to-face & Need internet & Privacy & Paying the cost to continue telemedicine services & Having difficulties related to audio echo & Having brief lag between auditory and visual images |

| | | | | | | | | | | | | | |
|---|---|---|---|---|---|---|---|---|---|---|---|---|---|
| Poon *et al.*[5] (2005) | China | Quantitative/ RCT | No academic | Clinic | AD (n=22) | Six weeks | P-P | N/M | VC / VTM / CVT / VTC | Feasibility & Comparing the effects of telecare and face-to-face therapy & Acceptability | Cost effectiveness & Patient/provider satisfaction | Reducing travel and saving time | Patients prefer face-to-face |
| Vestal *et al.*[6] (2006) | USA | Quantitative/ RCT | No academic | Clinic | AD mean age=73.9 (N=15 n=10) | N/M | P-P | N/M | VC / VTM / CVT / VTC | Comparing the effects of telecare and face-to-face therapy | Patient/provider satisfaction | Providing services in under-served rural areas & Reducing travel and saving time | Need internet & Presence of background noise |
| Cullum *et al.*[7] (2006) | USA | Quantitative/ Cross sectional | Academic | Home* | MCI Mean age=73 (n=14)<br>Moderate AD Mean age=73 (n=19) | Two months | P-P | N/M | VC / VTM / CVT / VTC | Feasibility | Cost effectiveness | N/M | Patients prefer face-to-face & Patients required assistance/caregiver |
| Loh *et al.*[8] (2007) | Australia | Quantitative/ RCT | Academic | Rural clinic | AD Mean age=79 (n=20)<br>Physician (control group n=8, intervention group n=2) | N/M | P-P | N/M | VC / VTM / CVT / VTC | Diagnosis | N/M | Providing services in under-served rural areas | Patients prefer face-to-face & Need internet & The telemedicine required that had good hearing and eyesight & Patients often have behavioral problems and they may not therefore be cooperative & Siting attentively in front of a television screen for at least an hour |
| Mundt *et al.*[9] (2007) | USA | Quantitative/ Cohort | Academic | Home | Individual>65 (n=36)<br>MCI>65 (n=37)<br>AD>65 (n=34) | 4, 12 and 20 weeks | P-P | N/M | Telephone-based | Monitoring | Cost effectiveness & Improving patient care | Helping to research | N/M |

| | | | | | | | | | | | | | |
|---|---|---|---|---|---|---|---|---|---|---|---|---|---|
| Smith *et al.*[10] (2007) | USA | Quantitative/ Case control | Academic | Home | Contact total (N=4472)<br>Phone contact (n=1855)<br>Video contact (n=2617) | six months | P-P | Social worker/care manager, Child | Other | Monitoring | Cost effectiveness & Decreasing concern/worry (lost or danger)/stress/anxiety / depression & Reducing medication delivery problems | Providing services in under-served rural areas & Training patients for self-administration & Involving clinicians | Patients required assistance/caregiver & Living in their own home or apartment & Turn off the monitor or camera by patient & Paying the cost to continue telemedicine services & Technical glitches & The equipment was a bit intrusive |
| Wray *et al.*[11] (2010) | USA | Quantitative/ RCT | Academic | Home | PwD, AD and Cg (N=1649, n=158) | 6-12 months | P-P & Cg-P | N/M | Telephone-based | Ancillary tools to empower PwD or Cg | Cost effectiveness | Training patients for self-administration & Reducing in the number of nursing home days | Patients required assistance/caregiver |
| Barton *et al.*[12] (2011) | USA | Quantitative/ Cross sectional | Academic | Rural clinic | Moderate AD Mean age=79.1 (n=19)<br>MCI Mean age=79.1 (n=2)<br>Individual Mean age=79.1 (n=1) | N/M | P-P | N/M | VC / VTM / CVT / VTC | Diagnosis | Cost effectiveness & Patient/provider satisfaction | Providing services in under-served rural areas & Reducing travel and saving time & Training caregivers | Patients prefer face-to-face & Security (HIPAA) & Patients required assistance/caregiver & Needing to room in clinic |
| Oderda *et al.*[13] (2011) | USA | Qualitative/ Description | Academic | Home | Clinical pharmacists (n=2)<br>PwD and AD | One year | P-P & Cg-P | N/M | Telephone-based | Developing | Cost effectiveness & Improving patient care | Reducing caregiver burden | N/M |

| | | | | | | | | | | | | | |
|---|---|---|---|---|---|---|---|---|---|---|---|---|---|
| Weiner *et al.*[14] (2011) | USA | Quantitative/ Non randomized trial | No academic | Clinic | PwD and AD (n=85) | 5 years | P-P | N/M | VC / VTM / CVT / VTC | Diagnosis & Management | Cost effectiveness & Increasing quality of Life | Providing services in under-served rural areas & Involving family | Patients prefer face-to-face & Needing to room in clinic & Some patients did not understand that they had seen a doctor |
| Wilz *et al.*[15] (2011) | Germany | Quantitative/ RCT | Academic | Home | Cg (N=343, n=229) | 2 years | Cg-P | N/M | Telephone-based | Effectiveness | N/M | Involving family & Increasing motivation | Patients required assistance/caregiver |
| Azad *et al.*[16] (2012) | Canada | Quantitative/ Cross sectional | No academic | Rural clinic | MCI or mild dementia and AD >=65 (n=99) | 4 years | P-P | N/M | VC / VTM / CVT / VTC | Follow-up | Patient/provider satisfaction & Improving patient care | Providing services in under-served rural areas & Reducing travel and saving time & Involving family & Increasing safety and risk management (e.g. prevent falls in night) | Patients prefer face-to-face & Patients required assistance/caregiver & Privacy & Needing to room in clinic & Technical glitches & Software errors & Increasing anxiety |
| Chou *et al.*[17] (2012) | Taiwan | Qualitative/ Description | Academic | Home | Cg Mean age=82.6 (n=30) | 6 month | Cg-P | Child, Spouses | Other | Ancillary tools to empower PwD or Cg & Evaluating the user friendliness, usefulness of telecare | Decreasing concern/worry (lost or danger)/stress/anxiety / depression & Improving patient care & | Reducing caregiver burden & Increasing safety and risk management (e.g. | Unfamiliar with technology |

| | | | | | | | | | | | | | |
|---|---|---|---|---|---|---|---|---|---|---|---|---|---|
| | | | | | | | | | | | Reducing uncertainty | prevent falls in night) & Usefulness, Ease of use | |
| Glueckauf *et al.*[18] (2012) | USA | Quantitative/ RCT | Academic | Home | Cg Mean age=60.89 (N=14, n=10, control group =5, intervention group =5) | 6 months | Cg-P | N/M | Telephone-based | Comparing the effects of telecare and face-to-face therapy | N/M | N/M | Patients prefer face-to-face |
| Martin-Khan *et al.*[19] (2012) | Australia | Quantitative/ Cohort | Academic | Clinic | PwD and AD >=50 (N=270, n=205)<br>Physician (n=2) | 2 years | P-P | N/M | VC / VTM / CVT / VTC | Validity a scale | Cost effectiveness & Patient/provider satisfaction | Providing services in under-served rural areas & Reducing travel and saving time & Helping to diagnosis of dementia | Patients prefer face-to-face & Need internet |
| Mitseva *et al.*[20] (2012) | Greece, Finland, Denmark, and UK | Quantitative/ Cross sectional | Academic | Home[&] | MCI or mild dementia (n=80)<br>Cg (N=7, control group = 45, intervention group = 26) | 15 months | P-P & Cg-P | Child, Spouses | Other | Ancillary tools to empower PwD or Cg | Patient/provider satisfaction & Increasing quality of Life & Improving patient care & Decreasing concern/worry (lost or danger)/stress/anxiety / depression | Reducing travel and saving time & Increasing safety and risk management (e.g. prevent falls in night), Accepting the technology and the services | Patients prefer face-to-face & Need to hardware (e.g. computer, laptop, microphone, webcam, sensor and …) at home & Need internet & Privacy |
| Parikh *et al.*[21] | USA | Quantitative/ | Academic | Room | Individual | 10-20 years | P-P | N/M | VC / VTM / CVT / | Acceptability | Patient/pro | N/M | Patients |

| | | | | | | | | | | | | | |
|---|---|---|---|---|---|---|---|---|---|---|---|---|---|
| (2013) | | RCT | | | (n=21)<br>AD (n=7)<br>MCI (n=12) | | | | VTC | | vider satisfactio n | | prefer face-to-face & Need to hardware (e.g. computer, laptop, microphone, webcam, sensor and …) at home & Privacy & The telemedicine required that had good hearing and eyesight |
| Martin *et al.*[22] (2013) | UK | Qualitative/ Description | Academic | Home[&] | PwD and AD (n=8) | 3 months | P-P | N/M | Other | Ancillary tools to empower PwD or Cg | Improving patient care | Increasi ng safety and risk manage ment (e.g. prevent falls in night) | Patients prefer face-to-face & Living in their own home or apartment |
| Tchalla *et al.*[23] (2013) | France | Quantitative/ RCT | No academic | Home | Mild and moderate AD >65 (N=96, intervention group=49, control group= 47) | One year | P-P | N/M | Telephone-based | Ancillary tools to empower PwD or Cg | N/M | Increasi ng safety and risk manage ment (e.g. prevent falls in night) | Need to hardware (e.g. computer, laptop, microphone, webcam, sensor and …) at home |
| Williams *et al.*[24] (2013) | USA | Quantitative/ RCT | Academic | Home[&] | Cg (N= N/M) | 3 months | Cg-P | N/M | VC / VTM / CVT / VTC | Monitoring | Increasing quality of Life | Providin g services in under-served rural areas | Need to hardware (e.g. computer, laptop, microphone, webcam, sensor and …) at home & Need internet & Security (HIPAA) & Privacy |
| Harrell *et al.*[25] (2014) | USA | Quantitative/ Cross sectional | No academic | Clinic | PwD, AD and Cg Mean age=74.88 (N=100, | 2 weeks | P-P | N/M | VC / VTM / CVT / VTC | Feasibility | N/M | Reducin g travel and saving time | Need to hardware (e.g. computer, laptop, |

| | | | | | | | | | | | | | |
|---|---|---|---|---|---|---|---|---|---|---|---|---|---|
| | | | | | n=31) | | | | | | | | microphone, webcam, sensor and …) at home |
| Jelcic *et al.*[26] (2014) | Italy | Quantitative/ RCT | No academic | Clinic | AD (N=38, n=27) | 3 months | P-P | N/M | VC / VTM / CVT / VTC | Feasibility & Efficacy | Cost effectiveness | Providing services in under-served rural areas & Increasing safety and risk management (e.g. prevent falls in night) | Patients prefer face-to-face |
| Cullum *et al.*[27] (2014) | USA | Quantitative/ Case-control | Academic | Clinic | PwD and AD Mean age=68.5 (n=202) | N/M | P-P | N/M | VC / VTM / CVT / VTC | Comparing the effects of telecare and face-to-face therapy | N/M | Providing services in under-served rural areas & Accepting the technology and the services | Patients prefer face-to-face & Need internet & Needing to room in clinic & The telemedicine required that had good hearing and eyesight |
| O'Connell *et al.*[28] (2014) | Canada | Qualitative/ Description | No academic | Home | Cg (N=10, n=7) | 18 months | Cg-P | Spouses | VC / VTM / CVT / VTC | Developing | N/M | Providing services in under-served rural areas & Reducing travel and saving time | Patients prefer face-to-face & Need internet & Security (HIPAA) & Technical problems related to the virtual nature of the connection & Presence of background noise |
| Catic *et al.*[29] (2014) | USA | Quantitative/ Cross sectional | No academic | Long-term care sites | AD Mean age=82 (N=47, n=39) | Median= 18 months | P-P | N/M | VC / VTM / CVT / VTC | Presenting the experiences and outcomes of designing and | Cost effectiveness & Improving clinical outcomes | N/M | Need to hardware (e.g. computer, laptop, microphone, |

| | | | | | | | | | | | | | |
|---|---|---|---|---|---|---|---|---|---|---|---|---|---|
| | | | | | | | | | | implementing an telecare | & Decreasing hospitalization and mortality | | webcam, sensor and …) at home & Need internet & Security (HIPAA) & Satisfying medical licensing requirements in some states that do not allow licensed physicians from other states to consult with their physicians |
| Schaller *et al.*[30] (2015) | Germany | Quantitative/ Cross sectional | Academic | Home | Cg (n=42) | 3 months | Cg-P | Child, Spouses | Other | Ancillary tools to empower PwD or Cg | Cost effectiveness & Decreasing concern/worry (lost or danger)/stress/anxiety / depression | Reducing travel and saving time & Reducing caregiver burden & Increasing motivation | Privacy |
| Cheong *et al.*[31] (2015) | South Korea | Quantitative/ RCT | Academic | Rural clinic | PwD and AD <=70 (N=442, n=427, control group =259, intervention group =168) | Five year | P-P | N/M | VC / VTM / CVT / VTC | Effectiveness | Patient/provider satisfaction | Accepting the technology and the services & Decreasing the disease progression in dementia patients in rural areas | Patients prefer face-to-face & Security (HIPAA) |
| Hattink *et al.*[32] (2015) | The Netherlands, Sweden, Italy, Malta, Romania, and | Quantitative/ RCT | Academic | Home | Cg (N=142, In the Netherlands=85 and in UK=57) | For 2 to 4 months | Cg-P | N/M | Other | Evaluating the user friendliness, usefulness of telecare | N/M | Usefulness & User friendliness | Need to hardware (e.g. computer, laptop, |

| | | | | | | | | | | | | | |
|---|---|---|---|---|---|---|---|---|---|---|---|---|---|
| | UK | | | | | | | | | | | | microphone, webcam, sensor and …) at home & Need internet & No having computer at home |
| Kim *et al.*[33] (2015) | South Korea | Quantitative/ RCT | Academic | Clinic | PwD and AD (N=188, control group =90, intervention group =98) | Five years | P-P | N/M | VC / VTM / CVT / VTC | Effectiveness | N/M | Providing services in under-served rural areas | Patients prefer face-to-face & Need to hardware (e.g. computer, laptop, microphone, webcam, sensor and …) at home |
| Pakrasi *et al.*[34] (2015) | UK and USA | Qualitative/ Description | No academic | Home | PwD and AD in UK (N=125 n=26)<br>Family in UK and USA (n=109) | Three years | P-P | N/M | VC / VTM / CVT / VTC | Ancillary tools to empower PwD or Cg | Cost effectiveness &Increasing quality of Life & Reducing Stigma | Providing services in under-served rural areas | N/M |
| Bowes *et al.*[35] (2016) | The Faroe Islands, Greenland, Sweden and Scotland | Mix method/ Cross sectional | Academic | Home | PwD and AD >60 and Cg (n=78) | N/M | P-P & Cg-P | N/M | Other | Developing & Evaluating telecare services | N/M | Reducing travel and saving time, facilitating and supporting local services for the patient | Living in their own home or apartment |
| Burton *et al.*[36] (2016) | Canada | Quantitative/ Cross sectional | Academic | Room[#] | MCI (n=5)<br>AD (n=8)<br>AD / VaD (n=2) | 3 years | P-P & Cg-P | N/M | VC / VTM / CVT / VTC | Developing | Patient/provider satisfaction | Increasing motivation | N/M |
| Lindauer *et al.*[37] (2017) | USA | Quantitative/ RCT | Academic | Home | PwD and AD Mean age=71.6 (n=33)<br>Cg Mean age=65.3 (n=33) | 2 weeks | P-P & Cg-P | N/M | VC / VTM / CVT / VTC | Feasibility & Reliability | Cost effectiveness | N/M | Patients prefer face-to-face & Need to hardware (e.g. computer, laptop, microphone, webcam, |

| | | | | | | | | | | | | | |
|---|---|---|---|---|---|---|---|---|---|---|---|---|---|
| | | | | | | | | | | | | | sensor and …) at home & Need internet & Security (HIPAA) & The telemedicine required that had good hearing and eyesight & Technical problems related to the virtual nature of the connection |
| Mavandadi *et al.*[38] (2017) | USA | Quantitative/ RCT | Academic | Home | Cg<br>Mean age=64 (n=440) | 3 and 6 months | Cg-P | N/M | Telephone-based | Ancillary tools to empower PwD or Cg | N/M | Providing services in under-served rural areas, Reducing caregiver burden | N/M |
| Tremont *et al.*[39] (2017) | Island | Quantitative/ RCT | Academic | Home | Cg<br>Mean age=62.7 (N=250 control group =133, intervention group =117) | >6 months | Cg-P | N/M | Telephone-based | Effectiveness | Cost effectiveness & Decreasing concern/worry (lost or danger)/stress/anxiety / depression & Decreasing hospitalization and mortality | N/M | N/M |
| Burton *et al.*[40] (2018) | Canada | Quantitative/ RCT | Academic | Room[#] | SCI (n=4)<br>AD (n=1) | > 8 weeks | Cg-P | N/M | VC / VTM / CVT / VTC | Feasibility & Acceptability | Increasing quality of Life | N/M | N/M |
| Dang *et al.*[41] (2018) | USA | Quantitative/ Cross | No academic | Clinic room | PwD and AD (n=94) | 19 months | P-P & Cg-P | N/M | VC / VTM / CVT / VTC | Feasibility & Acceptability | Patient/provider | Providing | N/M |

| | | | | | | | | | | | | | |
|---|---|---|---|---|---|---|---|---|---|---|---|---|---|
| | | sectional | | | Cg (n=41) | | | | | | satisfaction | services in under-served rural areas & Helping to diagnosis of dementia | |
| Powers *et al.*[42] (2018) | USA | Quantitative/ Cross sectional | No academic | Outpatient clinic | PwD and AD (n=36) | 3 years | P-P & Cg-P | Family members, Spouses | VC / VTM / CVT / VTC | Ancillary tools to empower PwD or Cg | Patient/provider satisfaction | Providing services in under-served rural areas & Reducing travel and saving time | Need to hardware (e.g. computer, laptop, microphone, webcam, sensor and …) at home & Security (HIPAA) & Increasing stress in caregiver |
| Lindauer *et al.*[43] (2018) | USA | Mix method/ Cross sectional | Academic | Home | Cg (n=20) | 2 months | Cg-P | N/M | VC / VTM / CVT / VTC | Feasibility & Efficacy & Examining cost of implementing a telecare | Cost effectiveness | Involving family & Training caregivers | Need to hardware (e.g. computer, laptop, microphone, webcam, sensor and …) at home & Need internet & Unfamiliar with technology & Increasing anxiety & Increasing stress in caregiver & Increasing Stigma |
| Carotenuto *et al.*[44] (2018) | Italy | Quantitative/ RCT | Academic | Hospital room | AD>50 (n=28) | 24 months | P-P & Cg-P | Child, Spouses | VC / VTM / CVT / VTC | Examining and assess a scale | N/M | Reducing travel and saving time | Patients prefer face-to-face & Need to hardware (e.g. computer, laptop, microphone, webcam, |

| | | | | | | | | | | | | | |
|---|---|---|---|---|---|---|---|---|---|---|---|---|---|
| | | | | | | | | | | | | | sensor and …) at home & Need internet & Security (HIPAA) & Patients required assistance/caregiver & The telemedicine required that had good hearing and eyesight |
| Chang *et al.*[45] (2018) | USA | Quantitative/ Observational | Academic | Rural clinic | PwD and AD (n=199) | One year | P-P & Cg-P | N/M | VC / VTM / CVT / VTC | Ancillary tools to empower PwD or Cg | Increasing quality of Life & Reducing medication delivery problems | N/M | Need to hardware (e.g. computer, laptop, microphone, webcam, sensor and …) at home & Security (HIPAA) & Needing to room in clinic |
| Griffith *et al.*[46] (2018) | USA | Quantitative/ Before-after | Academic | Home | Cg (N=64 n=57) | 6 weeks | Cg-P | Spouses | VC / VTM / CVT / VTC | Feasibility & Efficacy | Decreasing concern/worry (lost or danger)/stress/anxiety / depression | Providing services in under-served rural areas & Reducing caregiver burden & Training caregivers | Need to hardware (e.g. computer, laptop, microphone, webcam, sensor and …) at home & Need internet & Privacy |
| Wilz *et al.*[47] (2018) | Germany | Quantitative/ RCT | Academic | Home | Cg Mean age =64.10 (N=273, intervention group= 139, control group = 134) | 6 months | Cg-P | Child, Spouses | Telephone-based | Effectiveness | Patient/provider satisfaction & Decreasing concern/worry (lost or danger)/stress/anxiety / | Involving family | Patients prefer face-to-face |

| | | | | | | | | | | | | | |
|---|---|---|---|---|---|---|---|---|---|---|---|---|---|
| | | | | | | | | | | | depression & Improving behavioral symptoms patient | | |
| Wadsworth *et al.*[48] (2018) | USA | Quantitative/ RCT | Academic | N/M | PwD and AD (N=197, control group= 119, intervention group= 78) | 7 and 14 days | P-P | N/M | VC / VTM / CVT / VTC | Ancillary tools to empower PwD or Cg | N/M | N/M | Patients prefer face-to-face & Need to hardware (e.g. computer, laptop, microphone, webcam, sensor and …) at home & Need internet |
| Töpfer *et al.*[49] (2018) | Germany | Quantitative/ RCT | Academic | Home | Cg mean age =64.19 (N=273, intervention group = 139, control group = 134) | One year | Cg-P | N/M | Telephone-based | Ancillary tools to empower PwD or Cg | N/M | Involving family & Increasing motivation | N/M |
| Lindauer *et al.*[50] (2019) | USA | Quantitative/ Before-after | Academic | Home | Cg (N=20) | 2 months | Cg-P | Sibling, Spouses | VC / VTM / CVT / VTC | Efficacy | Cost effectiveness & Decreasing concern/worry (lost or danger)/stress/anxiety / depression | Reducing travel and saving time & Involving family & Reducing caregiver burden | Need to hardware (e.g. computer, laptop, microphone, webcam, sensor and …) at home & Need internet & Security (HIPAA) |
| Gustafson *et al.*[51] (2019) | USA | Quantitative/ RCT | Academic | Home | Cg (N=31) | 6 months | Cg-P | Child, Spouses | Other | Effectiveness | Decreasing concern/worry (lost or danger)/stress/anxiety / depression & Increasing quality of Life | Involving family & Reducing caregiver burden | Need to hardware (e.g. computer, laptop, microphone, webcam, sensor and …) at home & Turn off the monitor or camera by patient |

| | | | | | | | | | | | | | |
|---|---|---|---|---|---|---|---|---|---|---|---|---|---|
| Banbury *et al.*[52] (2019) | Australia | Quantitative/ RCT | Academic | Home* | Cg Mean age=63 (N=69) | 6 weeks | Cg-P | Child, Spouses | VC / VTM / CVT / VTC | Developing & Evaluating telecare services | N/M | N/M | Need internet & Needing to room in clinic |
| Williams *et al.*[53] (2019) | USA | Quantitative/ RCT | Academic | Home& | Cg ( N=84, intervention group =43, control group =41) | 3 months | Cg-P | Child, Spouses | VC / VTM / CVT / VTC | Ancillary tools to empower PwD or Cg | Patient/provider satisfaction & Decreasing concern/worry (lost or danger)/stress/anxiety / depression | Involving family & Reducing caregiver burden | Need to hardware (e.g. computer, laptop, microphone, webcam, sensor and …) at home & Need internet & Living in their own home or apartment |
| Thomas *et al.*[54] (2019) | USA | Qualitative/ Case study | Academic | Home | Cg (N= N/M) | 12-18 months | Cg-P | Spouses | VC / VTM / CVT / VTC | Comparing the effects of telecare and face-to-face therapy | Decreasing concern/worry (lost or danger)/stress/anxiety / depression | Reducing caregiver burden | Need to hardware (e.g. computer, laptop, microphone, webcam, sensor and …) at home |
| Mavandadi *et al.*[55] (2019) | USA | Quantitative/ RCT | No academic | Home | Cg mean age =83.1 (N=158, n=107) | >18 months | Cg-P | Spouses | Telephone-based | Examining and assess a scale | Patient/provider satisfaction & Increasing quality of Life | N/M | Patients required assistance/caregiver & Living in their own home or apartment |
| Moo *et al.*[56] (2020) | USA | Quantitative/ Non randomized trials | Academic | Home | Family mean age for patient=82 (N=222 control group =184, intervention group =38) | One year | Cg-P | Social worker/care manager, Child, Spouses | VC / VTM / CVT / VTC | Comparing the effects of telecare and face-to-face therapy | Patient/provider satisfaction & Decreasing concern/worry (lost or danger)/stress/anxiety / depression & Improving patient care | Reducing travel and saving time | Need to hardware (e.g. computer, laptop, microphone, webcam, sensor and …) at home & Need internet & Security (HIPAA) & Patients required assistance/caregiver |

| | | | | | | | | | | | | | |
|---|---|---|---|---|---|---|---|---|---|---|---|---|---|
| Laver *et al.*[57] (2020) | Australia | Quantitative/ RCT | Academic | Home | PwD and AD (N=63) | 4 months | P-P | N/M | VC / VTM / CVT / VTC | Comparing the effects of telehealth and face-to-face therapy | N/M | Reducin g travel | N/M |
| Williams *et al.*[58] (2021) | USA | Mix method/ RCT | Academic | Home | PwD and AD (N= 71)<br>Cg (N= 83) | 3 months | Cg-P | Child, Spouses | VC / VTM / CVT / VTC | Satisfaction, utilization, and feasibility of a telehealth intervention | Feasibility & Improving patient care | Involvin g family & | Privacy |
| Howard *et al.*[59] (2021) | UK | Quantitative/ RCT | Academic | Home | PwD and AD (N= 200) | 3 months | P-P | N/M | Other | The effectiveness and cost-effectiveness of assistive technology and telecare | Cost effectivene ss & effectivene ss | Involvin g clinician s | N/M |
| Di Lorito *et al.*[60] (2021) | UK | Qualitative/ Case study | Academic | Home | PwD and AD>=65 (N=5)<br>Cg (N= 10) | 4 months | Cg-P<br>P-P | Child, Spouses | VC / VTM / CVT / VTC | Tele-Rehabilitatio n for PwD during the COVID-19 pandemic | Time efficiency of sessions & enhancing participant s' motivation & caregivers' dementia awareness & therapists' creativity | Involvin g clinician s & Involvin g family | Patients required assistance/car egiver |

the United Kingdom (UK), the United States of America (USA), Mild Cognitive Impairment (MCI), People with Dementia (PwD), Caregiver (Cg), Alzheimer Disease (AD), Vascular Dementia (VaD), Subjective Cognitive Impairment (SCI), Patient-Physician (P-P), Caregiver- Physician (Cg-P), Video Conferencing (VC), Video Telemedicine (VTM), Clinical Video Telehealth (CVT), Video Telecommunication (VTM), and Not Mentioned (N/M)

[$] The year of publication of the articles in the table is from old to new

[*] Virtual room

[#] Room in university

[&] Smart or intelligence or expert home

## References


1. Harvey R, Roques PK, Fox NC, Rossor MN. CANDID--Counselling and Diagnosis in Dementia: a national telemedicine service supporting the care of younger patients with dementia. International journal of geriatric psychiatry. 1998;13(6):381-8. doi: 10.1002/(sici)1099-1166.
2. Lee JH, Kim JH, Jhoo JH, Lee KU, Kim KW, Lee DY, et al. A telemedicine system as a care modality for dementia patients in Korea. Alzheimer disease and associated disorders. 2000;14(2):94-101. doi: 10.1097/00002093-200004000-00007.

3. Mahoney DF, Tarlow BJ, Jones RN. Effects of an automated telephone support system on caregiver burden and anxiety: findings from the REACH for TLC intervention study. The Gerontologist. 2003;43(4):556-67. doi: 10.1093/geront/43.4.556.
4. Shores MM, Ryan-Dykes P, Williams RM, Mamerto B, Sadak T, Pascualy M, et al. Identifying undiagnosed dementia in residential care veterans: comparing telemedicine to in-person clinical examination. International journal of geriatric psychiatry. 2004;19(2):101-8. doi: 10.1002/gps.1029.
5. Poon P, Hui E, Dai D, Kwok T, Woo J. Cognitive intervention for community-dwelling older persons with memory problems: telemedicine versus face-to-face treatment. International journal of geriatric psychiatry. 2005;20(3):285-6. doi: 10.1002/gps.1282.
6. Vestal L, Smith-Olinde L, Hicks G, Hutton T, Hart Jr J. Efficacy of language assessment in Alzheimer’s disease: comparing in-person examination and telemedicine. Clinical interventions in aging. 2006;1(4):467. doi: 10.2147/ciia.2006.1.4.467.
7. Cullum CM, Weiner MF, Gehrmann HR, Hynan LS. Feasibility of telecognitive assessment in dementia. Assessment. 2006;13(4):385-90. doi: 10.1177/1073191106289065.
8. Loh P-k, Donaldson M, Flicker L, Maher S, Goldswain P. Development of a telemedicine protocol for the diagnosis of Alzheimer's disease. Journal of telemedicine and telecare. 2007;13(2):90-4. doi: 10.1258/135763307780096159.
9. Mundt JC, Kinoshita LM, Hsu S, Yesavage JA, Greist JH. Telephonic remote evaluation of neuropsychological deficits (TREND): longitudinal monitoring of elderly community-dwelling volunteers using touch-tone telephones. Alzheimer Disease & Associated Disorders. 2007;21(3):218-24. doi: 10.1097/WAD.0b013e31811ff2c9.
10. Smith GE, Lunde AM, Hathaway JC, Vickers KS. Telehealth home monitoring of solitary persons with mild dementia. American Journal of Alzheimer's Disease & Other Dementias®. 2007;22(1):20-6. doi: 10.1177/1533317506295888.
11. Wray LO, Shulan MD, Toseland RW, Freeman KE, Vásquez BE, Gao J. The effect of telephone support groups on costs of care for veterans with dementia. The Gerontologist. 2010;50(5):623-31. doi: 10.1093/geront/gnq040.
12. Barton C, Morris R, Rothlind J, Yaffe K. Video-telemedicine in a memory disorders clinic: evaluation and management of rural elders with cognitive impairment. Telemedicine journal and e-health : the official journal of the American Telemedicine Association. 2011;17(10):789-93. doi: 10.1089/tmj.2011.0083.
13. Oderda L, Holman C, Nichols B, Rupper R, Bair B, Hoffman M, et al. Pharmacist-managed telephone clinic review of antidementia medication effectiveness. The Consultant Pharmacist®. 2011;26(4):264-73. doi: 10.4140/TCP.n.2011.264.
14. Weiner MF, Rossetti HC, Harrah K. Videoconference diagnosis and management of Choctaw Indian dementia patients. Alzheimer's & Dementia. 2011;7(6):562-6. doi: 10.1016/j.jalz.2011.02.006.
15. Wilz G, Schinköthe D, Soellner R. Goal attainment and treatment compliance in a cognitive-behavioral telephone intervention for family caregivers of persons with dementia. GeroPsych. 2011. doi: 10.1024/1662-9647/a000043.
16. Azad N, Amos S, Milne K, Power B. Telemedicine in a rural memory disorder clinic-remote management of patients with dementia. Canadian geriatrics journal : CGJ. 2012;15(4):96-100. doi: 10.5770/cgj.15.28.
17. Chou HK, Yan SH, Lin IC, Tsai MT, Chen CC, Woung LC. A pilot study of the telecare medical support system as an intervention in dementia care: the views and experiences of primary caregivers. The journal of nursing research : JNR. 2012;20(3):169-80. doi: 10.1097/jnr.0b013e318263d916.
18. Glueckauf RL, Davis WS, Willis F, Sharma D, Gustafson DJ, Hayes J, et al. Telephone-based, cognitive-behavioral therapy for african american dementia caregivers with depression: Initial findings. Rehabilitation Psychology. 2012;57(2):124-39. doi: 10.1037/a0028688.
19. Martin-Khan M, Flicker L, Wootton R, Loh P-K, Edwards H, Varghese P, et al. The diagnostic accuracy of telegeriatrics for the diagnosis of dementia via video conferencing. Journal of the American Medical Directors Association. 2012;13(5):487. e19-. e24. doi: 10.1016/j.jamda.2012.03.004.
20. Mitseva A, Peterson CB, Karamberi C, Oikonomou LC, Ballis AV, Giannakakos C, et al. Gerontechnology: providing a helping hand when caring for cognitively impaired older adults—intermediate results from a controlled study on the satisfaction and acceptance of informal caregivers. Current gerontology and geriatrics research. 2012; 19(1):200-229. doi: 10.1155/2012/401705.
21. Parikh M, Grosch MC, Graham LL, Hynan LS, Weiner M, Shore JH, et al. Consumer acceptability of brief videoconference-based neuropsychological assessment in older individuals with and without cognitive impairment. The Clinical Neuropsychologist. 2013;27(5):808-17. doi: 10.1080/13854046.2013.791723.
22. Martin S, Augusto JC, McCullagh P, Carswell W, Zheng H, Wang H, et al. Participatory research to design a novel telehealth system to support the night-time needs of people with dementia: NOCTURNAL. International journal of environmental research and public health. 2013;10(12):6764-82. doi: 10.3390/ijerph10126764.
23. Tchalla AE, Lachal F, Cardinaud N, Saulnier I, Rialle V, Preux P-M, et al. Preventing and managing indoor falls with home-based technologies in mild and moderate Alzheimer's disease patients: pilot study in a community dwelling. Dementia and geriatric cognitive disorders. 2013;36(3-4):251-61. doi: 10.1159/000351863.
24. Williams K, Arthur A, Niedens M, Moushey L, Hutfles L. In-home monitoring support for dementia caregivers: a feasibility study. Clinical nursing research. 2013;22(2):139-50. doi: 10.1177/1054773812460545.

25. Harrell KM, Wilkins SS, Connor MK, Chodosh J. Telemedicine and the evaluation of cognitive impairment: the additive value of neuropsychological assessment. J Am Med Dir Assoc. 2014;15(8):600-6. doi: 10.1016/j.jamda.2014.04.015.
26. Jelcic N, Agostini M, Meneghello F, Bussè C, Parise S, Galano A, et al. Feasibility and efficacy of cognitive telerehabilitation in early Alzheimer's disease: a pilot study. Clinical interventions in aging. 2014;9:1605-11. doi: 10.2147/CIA.S68145.
27. Cullum CM, Hynan L, Grosch M, Parikh M, Weiner M. Teleneuropsychology: Evidence for video teleconference-based neuropsychological assessment. Journal of the International Neuropsychological Society: JINS. 2014;20(10):1028. doi: 10.1017/S1355617714000873.
28. O'Connell ME, Crossley M, Cammer A, Morgan D, Allingham W, Cheavins B, et al. Development and evaluation of a telehealth videoconferenced support group for rural spouses of individuals diagnosed with atypical early-onset dementias. Dementia. 2014;13(3):382-95. doi: 10.1177/1471301212474143.
29. Catic AG, Mattison MLP, Bakaev I, Morgan M, Monti SM, Lipsitz L. ECHO-AGE: An Innovative Model of Geriatric Care for Long-Term Care Residents With Dementia and Behavioral Issues. Journal of the American Medical Directors Association. 2014;15(12):938-42. doi: 10.1016/j.jamda.2014.08.014.
30. Schaller S, Marinova-Schmidt V, Gobin J, Criegee-Rieck M, Griebel L, Engel S, et al. Tailored e-Health services for the dementia care setting: a pilot study of 'eHealthMonitor'. BMC medical informatics and decision making. 2015;15(1):1-9. doi: 10.1186/s12911-015-0182-2.
31. Cheong CK, Lim KH, Jang JW, Jhoo JH. The effect of telemedicine on the duration of treatment in dementia patients. Journal of telemedicine and telecare. 2015;21(4):214-8. doi:10.1177/1357633X14566571.
32. Hattink B, Meiland F, Van Der Roest H, Kevern P, Abiuso F, Bengtsson J, et al. Web-based STAR E-learning course increases empathy and understanding in dementia caregivers: Results from a randomized controlled trial in the netherlands and the United Kingdom. Journal of medical Internet research. 2015;17(10). doi: 10.2196/jmir.4025.
33. Kim H, Jhoo JH, Jang JW. The effect of telemedicine on cognitive decline in patients with dementia. Journal of telemedicine and telecare. 2017;23(1):149-54. doi: 10.1177/1357633X15615049.
34. Pakrasi S, Burmeister O, Coppola J, McCallum T, Loeb G. Ethical telehealth design for users with dementia. Gerontechnology. 2015; 13(4), 383–387. doi: 10.4017/gt.2015.13.4.002.00.
35. Bowes A, Dawson A, McCabe L. RemoDem: Delivering support for people with dementia in remote areas. Dementia (London, England). 2018;17(3):297-314. doi: 10.1177/1471301216643848.
36. Burton R, O'Connell ME, Morgan DG. Exploring interest and goals for videoconferencing delivered cognitive rehabilitation with rural individuals with mild cognitive impairment or dementia. NeuroRehabilitation. 2016;39(2):329-42. doi: 10.3233/NRE-161364.
37. Lindauer A, Seelye A, Lyons B, Dodge HH, Mattek N, Mincks K, et al. Dementia care comes home: patient and caregiver assessment via telemedicine. The Gerontologist. 2017;57(5):e85-e93. doi: 10.1093/geront/gnw206.
38. Mavandadi S, Wray LO, DiFilippo S, Streim J, Oslin D. Evaluation of a telephone-delivered, community-based collaborative care management program for caregivers of older adults with dementia. The American Journal of Geriatric Psychiatry. 2017;25(9):1019-28. doi: 10.1016/j.jagp.2017.03.015.
39. Tremont G, Davis JD, Ott BR, Galioto R, Crook C, Papandonatos GD, et al. Randomized trial of the family intervention: telephone tracking—caregiver for dementia caregivers: use of community and healthcare resources. Journal of the American Geriatrics Society. 2017;65(5):924-30. doi: 10.1111/jgs.14684.
40. Burton RL, O'Connell ME. Telehealth rehabilitation for cognitive impairment: randomized controlled feasibility trial. JMIR research protocols. 2018;7(2):e43. doi: 10.2196/resprot.9420.
41. Dang S, Gomez-Orozco CA, van Zuilen MH, Levis S. Providing Dementia Consultations to Veterans Using Clinical Video Telehealth: Results from a Clinical Demonstration Project. Telemedicine journal and e-health : the official journal of the American Telemedicine Association. 2018;24(3):203-9. doi: 10.1089/tmj.2017.0089.
42. Powers JS, Buckner J. Reaching out to rural caregivers and veterans with dementia utilizing clinical video-telehealth. Geriatrics. 2018;3(2):29. doi: 10.3390/geriatrics3020029.
43. Lindauer A, Croff R, Mincks K, Mattek N, Shofner S, Bouranis N, et al. "It Took the Stress out of Getting Help": The STAR-C-Telemedicine Mixed Methods Pilot. Care weekly. 2018;2:7. doi: 10.14283/cw.2018.4.
44. Carotenuto A, Rea R, Traini E, Ricci G, Fasanaro AM, Amenta F. Cognitive Assessment of Patients With Alzheimer's Disease by Telemedicine: Pilot Study. JMIR mental health. 2018;5(2):e31. doi: 10.2196/mental.8097.
45. Chang W, Homer M, Rossi MI. Use of Clinical Video Telehealth as a Tool for Optimizing Medications for Rural Older Veterans with Dementia. Geriatrics (Basel, Switzerland). 2018;3(3). doi: 10.3390/geriatrics3030044.
46. Griffiths PC, Kovaleva M, Higgins M, Langston AH, Hepburn K. Tele-Savvy: An Online Program for Dementia Caregivers. American journal of Alzheimer's disease and other dementias. 2018;33(5):269-76. doi: 10.1177/1533317518755331.

47. Wilz G, Reder M, Meichsner F, Soellner R. The Tele. TAnDem intervention: Telephone-based CBT for family caregivers of people with dementia. The Gerontologist. 2018;58(2):e118-e29. doi: 10.1093/geront/gnx183.
48. Wadsworth HE, Dhima K, Womack KB, Hart Jr J, Weiner MF, Hynan LS, et al. Validity of teleneuropsychological assessment in older patients with cognitive disorders. Archives of Clinical Neuropsychology. 2018;33(8):1040-5. doi: 10.1093/arclin/acx140.
49. Töpfer NF, Wilz G. Tele. TAnDem increases the psychosocial resource utilization of dementia caregivers. GeroPsych. 2018; 31(4): 173–183. doi: 10.1024/1662-9647/a000197.
50. Lindauer A, McKenzie G, LaFazia D, McNeill L, Mincks K, Spoden N, et al. Using technology to facilitate fidelity assessments: The Tele-STAR caregiver intervention. Journal of medical Internet research. 2019;21(5):e13599. doi: 10.2196/13599.
51. Gustafson DH, Gustafson DH, Cody OJ, Chih MY, Johnston DC, Asthana S. Pilot Test of a Computer-Based System to Help Family Caregivers of Dementia Patients. Journal of Alzheimer's disease : JAD. 2019;70(2):541-52. doi: 10.3233/JAD-190052.
52. Banbury A, Parkinson L, Gordon S, Wood D. Implementing a peer-support programme by group videoconferencing for isolated carers of people with dementia. Journal of telemedicine and telecare. 2019;25(9):572-7. doi: 10.1177/1357633X19873793.
53. Williams KN, Perkhounkova Y, Shaw CA, Hein M, Vidoni ED, Coleman CK. Supporting family caregivers with technology for dementia home care: A randomized controlled trial. Innovation in Aging. 2019;3(3):igz037. doi: 10.1093/geroni/igz037.
54. Thomas NW, Lindauer A, Kaye J. EVALUATE-AD and Tele-STAR: novel methodologies for assessment of caregiver burden in a telehealth caregiver intervention–a case study. Dementia and Geriatric Cognitive Disorders. 2019;47(3):176-84. doi: 10.1159/000497805.
55. Mavandadi S, Wray LO, Toseland RW. Measuring Self-Appraised Changes following Participation in an Intervention for Caregivers of Individuals with Dementia. Journal of gerontological social work. 2019;62(3):324-37. doi: 10.1080/01634372.2018.1556767.
56. Moo LR, Gately ME, Jafri Z, Shirk SD. Home-based video telemedicine for dementia management. Clinical gerontologist. 2020;43(2):193-203. doi: 10.1080/07317115.2019.1655510.
57. Laver K, Liu E, Clemson L, Davies O, Gray L, Gitlin LN, et al. Does telehealth delivery of a dyadic dementia care program provide a noninferior alternative to face-to-face delivery of the same program? A randomized, controlled trial. The American Journal of Geriatric Psychiatry. 2020;28(6):673-82. doi: 10.1016/j.jagp.2020.02.009.
58. Williams KN, Shaw CA, Perkhounkova Y, Hein M, Coleman CK. Satisfaction, utilization, and feasibility of a telehealth intervention for in-home dementia care support: A mixed methods study. Dementia. 2021;20(5):1565-85. doi: 10.1177/1471301220957905.
59. Howard R, Gathercole R, Bradley R, Harper E, Davis L, Pank L, et al. The effectiveness and cost-effectiveness of assistive technology and telecare for independent living in dementia: a randomised controlled trial. Age and ageing. 2021;50(3):882-90. doi: 10.1093/ageing/afaa284.
60. Di Lorito C, Duff C, Rogers C, Tuxworth J, Bell J, Fothergill R, et al. Tele-Rehabilitation for People with Dementia during the COVID-19 Pandemic: A Case-Study from England. International Journal of Environmental Research and Public Health. 2021;18(4):1717. doi: 10.3390/ijerph18041717.
